# Hydrothermal Synthesis of β-Nb_2_ZnO_6_ Nanoparticles for Photocatalytic Degradation of Methyl Orange and Cytotoxicity Study

**DOI:** 10.3390/ijms23094777

**Published:** 2022-04-26

**Authors:** Sarah Ameen Almofty, Muhammad Nawaz, Faiza Qureshi, Rayanah Al-Mutairi

**Affiliations:** 1 Department of Stem Cell Research, Institute for Research and Medical Consultations (IRMC), Imam Abdulrahman Bin Faisal University, P.O. Box 1982, Dammam 31441, Saudi Arabia; 2 Department of Nano-Medicine Research, Institute for Research and Medical Consultations (IRMC), Imam Abdulrahman Bin Faisal University, P.O. Box 1982, Dammam 31441, Saudi Arabia; frqureshi@iau.edu.sa (F.Q.); 2220500130@iau.edu.sa (R.A.-M.); 3 Deanship of Scientific Research, Imam Abdulrahman Bin Faisal University, P.O. Box 1982, Dammam 31441, Saudi Arabia

**Keywords:** β-Nb_2_ZnO_6_, photocatalysis, cytotoxicity, zeta potential, methyl orange, hydrothermal

## Abstract

β-Nb_2_ZnO_6_ nanoparticles were synthesized by a hydrothermal process and calcined at two temperatures, 500 °C and 700 °C, and assigned as **A** and **B,** respectively. X-ray diffraction, together with transmission electron microscopy, revealed that the β-Nb_2_ZnO_6_ nanoparticles calcined at 700 °C (**B**) were more crystalline than the β-Nb_2_ZnO_6_ calcined at 500 °C (**A**) with both types of nanoparticles having an average size of approximately 100 nm. The physiochemical, photocatalytic, and cytotoxic activities of both types of β-Nb_2_ZnO_6_ nanoparticles (**A** and **B**) were examined. Interestingly, the photodegradation of methyl orange, used as a standard for environmental pollutants, was faster in the presence of the β-Nb_2_ZnO_6_ nanoparticles calcined at 500 °C (**A**) than in the presence of those calcined at 700 °C (**B**). Moreover, the cytotoxicity was evaluated against different types of cancer cells and the results indicated that both types of β-Nb_2_ZnO_6_ nanoparticles (**A** and **B**) exhibited high cytotoxicity against MCF-7 and HCT116 cells but low cytotoxicity against HeLa cells after 24 and 48 h of treatment. Overall, both products expressed similar EC_50_ values on tested cell lines and high cytotoxicity after 72 h of treatment. As a photocatalyst, β-Nb_2_ZnO_6_ nanoparticles (**A**) could be utilized in different applications including the purification of the environment and water from specific pollutants. Further biological studies are required to determine the other potential impacts of utilizing β-Nb_2_ZnO_6_ nanoparticles in the biomedical application field.

## 1. Introduction

Nanotechnology has inspired the design of diverse nanoscale materials to fulfill the growing material requirements for environmental, biological, and medical applications [1,2,3,4,5,6]. Environmental pollution has become a serious global concern due to the rapid increase in population and industries. Most industrial wastes contain high concentrations of toxic substances that accumulate and negatively affect living creatures and the environment [7,8,9,10]. Therefore, designing nano-photocatalysts is needed to develop stable and effective photocatalysts for different types of organic pollutants. Recently, semiconductor photocatalysts have been extensively used for the purification of organic pollutants in water. TiO_2_, for example, is used as a catalyst for the photodegradation of organic pollutants, but with customized application only as it has a wide band gap, poor catalyst recovery, and a tendency to agglomerate [11,12]. Previously, we reported the catalytic degradation of volatile chlorinated compounds by heterostructured nanoparticles which showed enhanced catalytic activity [8,13,14,15,16,17,18]. Nanomaterials have also been used in the field of medicine and biology to significantly improve traditional diagnostic and therapeutic techniques [19,20,21,22]. They are also used as a powerful tool for the exploration and understanding of physiological processes [23,24,25,26] and offer many advantages as they have unique physiochemical attributes, including a large surface area compared to bulk biomolecules, and a stable shape and size which makes them interesting. Nanomaterials are typically smaller than host cells and can enter the host cells easily by cellular endocytosis or cell membrane penetration [27]. Several nanomaterials, such as titanium oxide, silica oxide, gold, copper oxide, copper sulfide, zinc sulfide/bismuth sulfide carbon nanotubes, silver oxide, and carbon-based materials, have been reported for their efficiency in biomedical and photocatalytic applications [28,29,30,31]. Interestingly, the composition of zinc incorporated niobium oxide exhibited antibacterial potential against *Staphylococcus aureus* and *Escherichia coli* bacteria [32], suggesting that this composition might have other potential activities yet to be investigated. Zinc oxide is considered an important semiconductor due to its stability, low cost, and excellent electron mobility, but its practical use in photocatalysis is limited due to its wide band gap (3.3 eV) and rapidly recombining electron-hole pairs [29,33]. Several methods have been employed to reduce the band gap such as metal/element doping, heterostructure engineering/construction, and controlling the morphology. Utilizing these strategies, some zinc oxide-based materials such as BioI/ZnO [34], ZnO@SiO_2_ [35], ZnO/CdSe [36], Ag/ZnO@C [37], and Ag_3_PO_4_/ZnO [38] have been synthesized successfully for efficient photocatalysis application.

Herein, we report the synthesis of β-Nb_2_ZnO_6_ nanoparticles (calcined at two different temperatures, 500 °C and 700 °C, designated as **A** and **B**, respectively, to reduce the recombination rate for electron-hole pairs and enhance the nanoparticles’ photocatalytic activities [29,39]. The prepared β-Nb_2_ZnO_6_ nanoparticles were distinguished by X-ray diffraction (XRD), transmission electron microscopy (TEM), BET surface analysis, zeta potential measurement, and diffuse reflectance- spectroscopy. The present work is designed to study (1) the potential photocatalytic activity of β-Nb_2_ZnO_6_ (**A**) and (**B**) nanoparticles by evaluating the photocatalytic degradation of methyl orange and (2) to determine the cytotoxicity of β-Nb_2_ZnO_6_ nanoparticles by measuring the cell viability of treated cancer cells to obtain the EC_50_ values.

## 2. Results and Discussion

### 2.1. Characterization of β-ZnNb_2_O_6_ Nanoparticles

**Figure 1:**
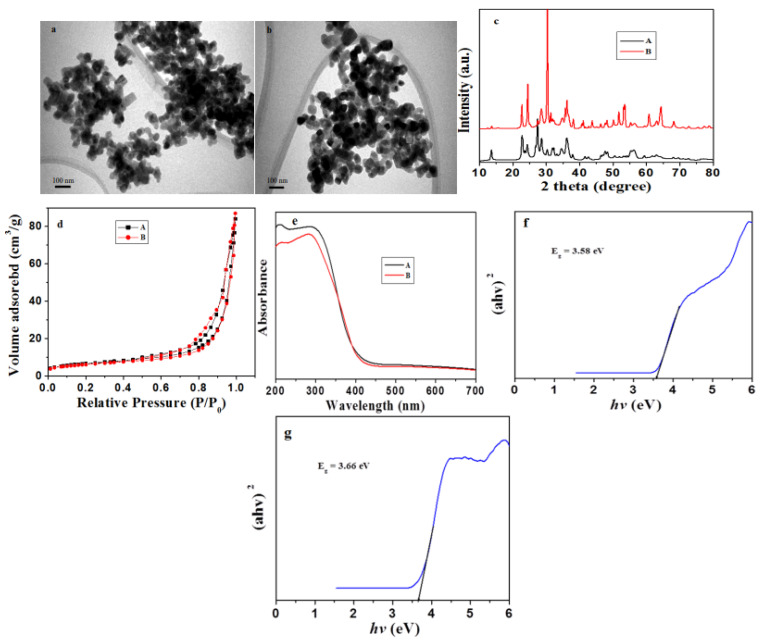
Physical characterization of the β-Nb_2_ZnO_6_ nanoparticles. TEM images of β-Nb_2_ZnO_6_ nanoparticles calcined at 500 °C (**A**) and 700 °C (**B**) (**a**,**b**), XRD pattern of β-Nb_2_ZnO_6_ (**A** and **B**) (**c**), N2 adsorption-desorption isotherm of β-Nb_2_ZnO_6_ (**A** and **B**) (**d**), UV-Vis spectra of β-Nb_2_ZnO_6_ (**A** and **B**) (**e**), Tauc plots of β-Nb_2_ZnO_6_ (**A** and **B**) (**f**,**g**).

The morphology of the β-Nb_2_ZnO_6_ (**A**) and (**B**) nanoparticles was determined by TEM. Figure 1a,b displays representative images of β-Nb_2_ZnO_6_ nanoparticles (**A** and **B**) calcined at 500 °C (**A**) and 700 °C (**B**), respectively. The average size of the β-Nb_2_ZnO_6_ (**A**) and (**B**) nanoparticles was 100 nm with nanoplate-like morphology. XRD was employed to find out the crystal structure of the β-Nb_2_ZnO_6_ (**A**) and (**B**) nanoparticles. Figure 1c shows the XRD configuration of β-Nb_2_ZnO_6_ calcined at 500 °C (**A**) and 700 °C (**B**). The results indicate that β-Nb_2_ZnO_6_ nanoparticles calcined at 700 °C (**B**) have more crystalline features than β-Nb_2_ZnO_6_ calcined at 500 °C (**A**). This further suggested that crystallinity can be achieved by calcining the sample at a high temperature. The peaks are well-matched with ICDD card no. 00-028-1477 with an unknown crystal system.

N_2_-adsorption-desorption isotherm examination was completed to review the pore size and surface area of the β-Nb_2_ZnO_6_ nanoparticles. Figure 1d shows the N_2_ sorption isotherms and porosity of the β-Nb_2_ZnO_6_ nanoparticles. The typical type-IV isotherm with a narrow H3-type hysteresis loop in Figure 1e suggested that the particles were mesoporous [40]. The BET surface area of β-Nb_2_ZnO_6_ calcined at 500 °C (**A**) was calculated to be 22.67 m^2^/g (pore size: 18.95 nm; pore volume 0.130 cm^3^/g), while the sample calcined at 700 °C (**B**)was found to have a surface area of 20.81 m^2^/g (pore size: 19.06 nm; pore volume 0.135 cm^3^/g). The slightly smaller surface area of β-Nb_2_ZnO_6_ nanoparticles calcined at 700 °C (**B**) is due to the improvement in the crystalline structure of the β-Nb_2_ZnO_6_ nanoparticles at high temperature.

Figure 1e displays the UV-visible absorption spectrum of β-Nb_2_ZnO_6_ nanoparticles (**A** and **B**) recorded in the range of 200–700 nm. The band gap can be calculated by the Tauc formula:

(α hv)^2/n^ =A (hv − E_g_),

where α represents absorption coefficient, h the Plank’s constant, v the light frequency, A an energy independent proportionality constant characteristic of the material, and E_g_ the optical band gap. The exponent *n* defines the nature of the optical transitions. The direct transition band gaps were determined from the Tauc plots of (α hv)^2^ vs. hv, (Figure 1f,g). The optical band gap E_g_ values for β-Nb_2_ZnO_6_ nanoparticles (**A** and **B**) were 3.58 and 3.66 eV, respectively.

**Figure 2:**
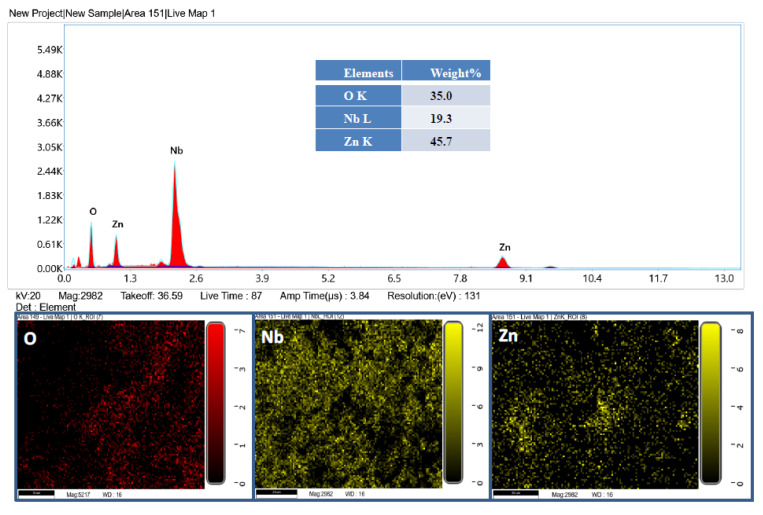
EDX spectrum and EDX mapping of β-Nb_2_ZnO_6_ nanoparticles (**A**).

The composition of the product was further confirmed by EDX analysis. The represented EDX spectrum and elemental mapping are shown in Figure 2. It is clear from Figure 2 that the product contains Nb, Zn, and O. K and L correspond to the amount of energy possessed by the X-ray emitted by an electron in the K and L shells, respectively.

**Figure 3:**
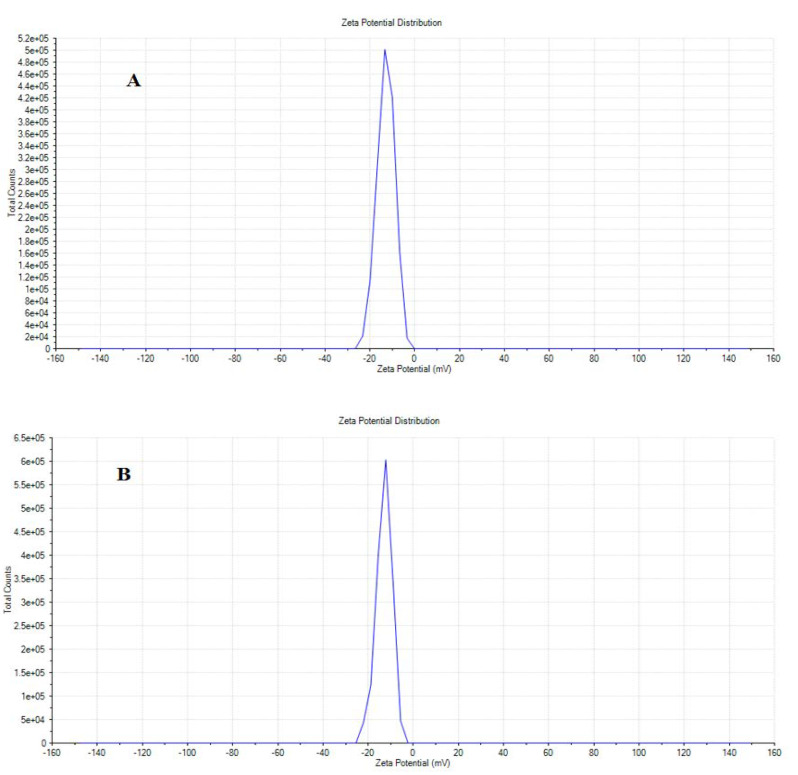
Zeta potential of β-Nb_2_ZnO_6_ nanoparticles (**A** and **B**).

Zeta potential is an imperative technique for determining the surface charge and stability of nanoparticles and can tell us the state of nanoparticle surface nature. The zeta potential of β-Nb_2_ZnO_6_ nanoparticles (**A** and **B**) is shown in Figure 3; both β-Nb_2_ZnO_6_ nanoparticles (**A** and **B**) have a less variation in the zeta potential and it was observed at −12.9 and −13 mV, respectively, while the polydispersity index (PDI) of β-Nb_2_ZnO_6_ nanoparticles (**B**) (PDI: 0.518) was slightly decreased as compared to β-Nb_2_ZnO_6_ nanoparticles (**A**) (PDI: 0.690). Similarly, the particle size distribution of β-Nb_2_ZnO_6_ nanoparticles (**A** and **B**) was observed at 517 and 453 nm, respectively. Results indicated almost similar stability of β-Nb_2_ZnO_6_ nanoparticles (**A** and **B**) in the deionized water.

### 2.2. Photocatalytic Activity of β-Nb_2_ZnO_6_ Nanoparticles

**Figure 4:**
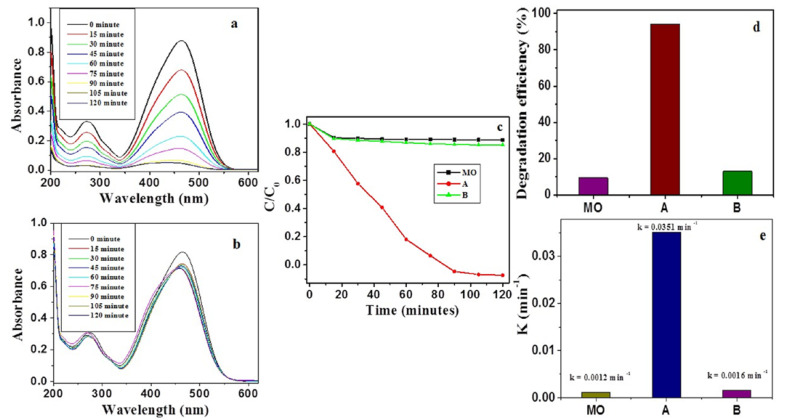
Changes in the UV-Vis absorption spectrum of methyl orange with 50 mg β-Nb_2_ZnO_6_ (**A** and **B**) after different irradiation times (**a**,**b**), photocatalytic activities of β-Nb_2_ZnO_6_ nanoparticles (**A** and **B**) for methyl orange degradation over 120 min (**c**), degradation efficiency of β-Nb_2_ZnO_6_ nanoparticles (**A** and **B**) (**d**) comparison of rate constants (**e**).

The photodegradation potential of β-Nb_2_ZnO_6_ nanoparticles (**A** and **B**) was tested using methyl orange as a typical environmental pollutant under UV-Vis light irradiation. As evident in Figure 4a, the absorption peak at 465 nm, which indicated the presence of methyl orange, decreased in the presence of β-Nb_2_ZnO_6_ nanoparticles (**A**) with increased reaction time. However, for sample B, the decrease in the absorption peak at 465 nm was small, even after long reaction times (Figure 4b). The photodegradation of methyl orange proceeded faster with β-Nb_2_ZnO_6_ nanoparticles calcined at 500 °C (**A**) (Figure 4c) compared to β-Nb_2_ZnO_6_ nanoparticles calcined at 700 °C (**B**). The photocatalytic degradation of methyl orange without β-Nb_2_ZnO_6_ nanoparticles was also studied; these results indicated that there was no degradation of methyl orange without β-Nb_2_ZnO_6_ nanoparticles, demonstrating that photocatalytic degradation of methyl orange can be achieved with β-Nb_2_ZnO_6_ nanoparticles (**A**) (Figure 4c). The photocatalytic efficiency of β-Nb_2_ZnO_6_ nanoparticles (**A**) was higher as compared to MO and β-Nb_2_ZnO_6_ nanoparticles (**B**) (Figure 4d). The high photocatalytic activity of the β-Nb_2_ZnO_6_ nanoparticles (**A**) could be credited to the highly effective separation of carrier charges as well as the slowed recombination of electron-hole pairs during the photocatalysis process causing high photocatalytic activity [41,42]. It has been noticed that various constraints such as pore dimension and formation and surface features and crystallinity affect the photocatalytic ability. The lesser photocatalytic ability of sample B may be caused by a decline in surface area, destruction of pores at high temperature, crystal growth, and shielding of active sites on the surface of sample B. This may result in a decrease in surface-active sites on the surface and a reduction in charge separation/transfer, which in turn causes low photocatalytic activity [40]. Furthermore, the kinetics of methyl orange degradation was evaluated to establish the rate constants and was found to follow pseudo-first order reactions:

ln(C_0_/C) = k t

where C_0_ and C are the initial and the time-dependent concentrations of methyl orange, t is the time (minutes) and k is the rate constant (min^−1^). As it is clear from Figure 4e), β-Nb_2_ZnO_6_ nanoparticles (A) revealed a high rate constant (0.0351 min^−1^) in the degradation of methyl orange as compared to β-Nb_2_ZnO_6_ nanoparticles (B) and blank methyl orange.

**Figure 5:**
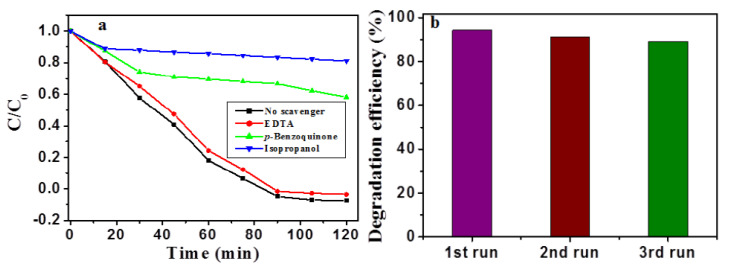
Degradation profile of methyl orange in the presence of scavengers (**a**); reusability of β-Nb_2_ZnO_6_ nanoparticles (**A**) after 3 runs (**b**).

Photocatalysis experiments were further conducted to understand the role of reactive species during the photocatalysis process. Scavenging agents such as isopropanol, *p*-benzoquinone, and ethylenediaminetetraacetic acid (EDTA) in the presence of β-Nb_2_ZnO_6_ nanoparticles (**A**) were used applying the same reaction conditions (Figure 5a). It was perceived that when EDTA was introduced in the reaction, there was a slight decline in the photocatalytic activity, suggesting the limiting role of the hole (h^+^) in photocatalysis. Additionally, when *p*-benzoquinone was used in the reaction, there was more decline in the photocatalytic activity, indicating that reactive superoxide radical (•O_2_^−^) performs an important role in photocatalysis process. Similarly, an isopropanol addition in the reaction resulted in a further decline in the photocatalytic activity, signifying that hydroxyl (•OH) plays a significant role in the photocatalytic degradation of methyl orange [43].

The recycling/reusability of a photocatalyst is important in the photocatalysis process, so we have tested the stability of β-Nb_2_ZnO_6_ nanoparticles (**A**) by recycling experiments. The same experimental conditions were used as in the photocatalytic experiment, except that after each run β-Nb_2_ZnO_6_ was separated, and fresh methyl orange was used. We performed three recycling experiments to check the stability of the β-Nb_2_ZnO_6_ nanoparticles (**A**). As it is clear from Figure 5b, the differences in photocatalytic activity after each recycling were minor, indicating the stability of β-Nb_2_ZnO_6_ nanoparticles.

**Figure 6:**
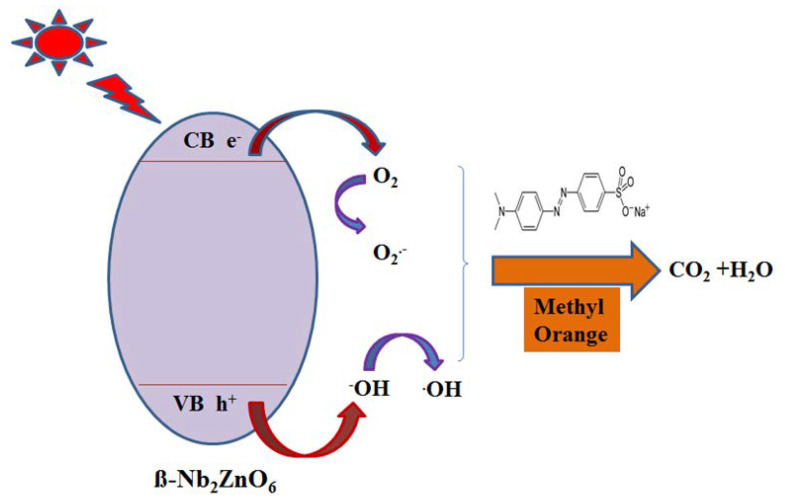
Schematic illustration of photocatalytic activity of β-Nb_2_ZnO_6_ for the degradation of methyl orange.

The mechanism of charge transfer on β-Nb_2_ZnO_6_ nanoparticles is illustrated in Figure 6, that is, the generation of electron-hole pairs (e^−^/h^+^), under UV light irradiation, in the conduction and valence bands of β-Nb_2_ZnO_6_ nanoparticles, respectively. The electrons in the conduction band of β-Nb_2_ZnO_6_ nanoparticles reduce the molecular oxygen to •O_2_^−^ and holes on the valence band interact with ^−^OH to produce •OH radicals, triggering the reduction and degradation of methyl orange [18].

### 2.3. Cytotoxicity of β-Nb_2_ZnO_6_ Nanoparticles

The cytotoxicity of β-Nb_2_ZnO_6_ nanoparticles had been recorded by estimating the cell viability via MTT assay [17,18]. MCF-7, HCT116, and HeLa cells were treated with pre-determined concentrations (1, 0.5, 0.25, and 0.125 mg/mL) of β-Nb_2_ZnO_6_ nanoparticles (**A**) and (**B**) for 24, 48, and 72h, followed by the cells incubating in an MTT solution, which utilizes a formazan, colorimetric reduction of tetrazolium salt (3-(4,5-dimethylthiazol-2-yl)-2,5-diphenyltetrazolium bromide) by living cells, producing purple crystals which, after dissolving in DMSO, were assayed at 570 nm on Elisa microplate reader.

**Figure 7:**
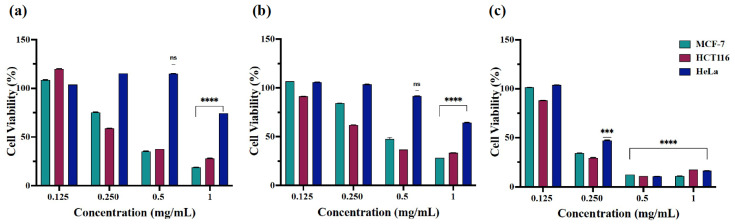
Cell viability (%) of MCF-7, HCT116 and HeLa cells treated with β-Nb_2_ZnO_6_ nanoparticles (**A**). The graph shows the cytotoxic effect of β-Nb_2_ZnO_6_ nanoparticles (**A**) as highly significant (****) when compared with untreated (control) cells, (**a**) treatment for 24 h, (**b**) 48 h and (**c**) 72 h. *P* value is set at <0.05 (* if *P* ≤ 0.05, ** if *P* ≤ 0.01, *** if *P* ≤ 0.001, **** if *P* ≤ 0.0001).

**Figure 8:**
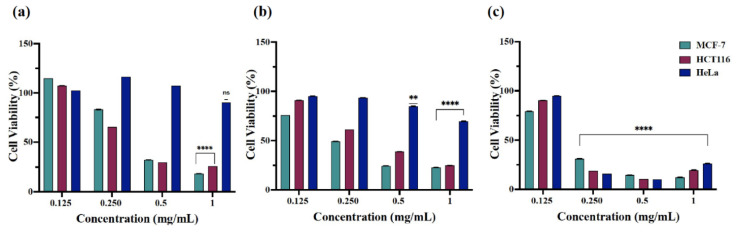
Cell viability (%) of MCF-7, HCT116 and HeLa cells treated with β-Nb_2_ZnO_6_ nanoparticles (**B**). The graph shows the cytotoxic effect of β-Nb_2_ZnO_6_ (**B**) nanoparticles as highly significant (****) when compared with untreated (control) cells, (**a**) treatment for 24 h, (**b**) 48 h and (**c**) 72 h. *P* value is set at <0.05 (* if *P* ≤ 0.05, ** if *P* ≤ 0.01, *** if *P* ≤ 0.001, **** if *P* ≤ 0.0001).

**Figure 9:**
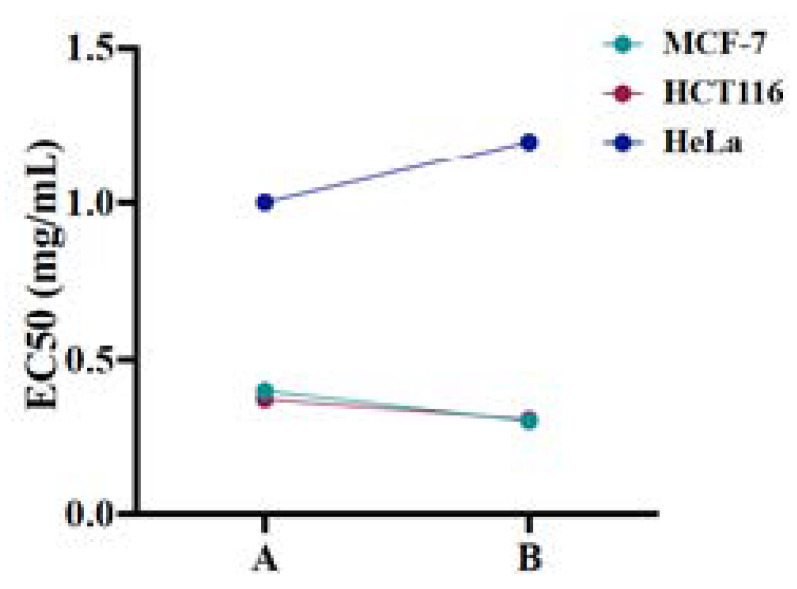
EC_50_ values of β-Nb_2_ZnO_6_ nanoparticles (**A**) and (**B**). Half maximal effective concentration (EC_50_) of β-Nb_2_ZnO_6_ (**A**) and (**B**) nanoparticles were determined for each cell line.

The assay exposed the increased cytotoxic potential of nanoparticles A at 0.5 and 1 mg/mL against MCF-7 and HCT116 cells with 30–47% cell viability and over 50% cell viability at the reduced nanoparticle concentrations following 24 and 48 h of treatment (Figure 7a,b). The cell viability of HeLa cells with nanoparticles (**A**) and (**B**) displayed less disparity among the used concentrations with the average cell viability being over 50% after 24 and 48 h of treatment. Interestingly, (**A**) and (**B**) exhibited increased cytotoxic action towards MCF-7, HCT116, and HeLa cells after 72 h of treatment at 0.250 mg/mL (Figure 7c and Figure 8c). However, the concentration effective for half-maximal response (EC_50_) values were estimated for (**A**) and (**B**) nanoparticles in each cell line after obtaining the average of cell viability% taken from the tested time points as EC_50_ = 0.401 mg/mL (**A**) and 0.305 mg/mL (**B**) for MCF-7 cells, 0.375 mg/mL (**A**) and 0.311 mg/mL (**B**) for HCT116 cells, and 1 mg/mL (**A**) and 1.2 mg/mL (**B**) for HeLa cells (Figure 9). Conclusively, the studies showed high cytotoxic action of β-Nb_2_ZnO_6_ nanoparticles (**A**) and (**B**) for MCF-7 and HCT116 cells and low cytotoxic action for HeLa cells, for which there is a need to explore further biomolecular assays to ascertain reasons for variable cytotoxicity. The present data, however, confirms that β-Nb_2_ZnO_6_ (**A**) and (**B**) have a close cytotoxic activity and further optimization is required for the enhancement of biocompatibility of β-Nb_2_ZnO_6_ for large-scale utilization.

### 2.4. Imaging of Nb_2_ZnO_6_ Nanoparticles Treated Cells

**Figure 10:**
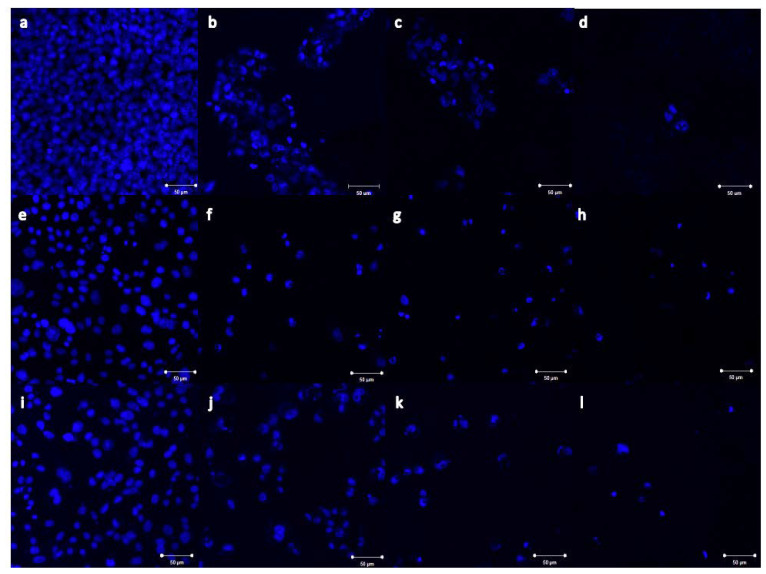
Confocal microscopic images of MCF-7, HCT116 and HeLa cells treated with β-Nb_2_ZnO_6_ nanoparticles (**A**). The cells were treated for 48 h with 0.25, 0.5, and 1 mg/mL of β-Nb_2_ZnO_6_ (**A**) nanoparticles. (**a**) Untreated MCF-7 cells, (**b**–**d**) MCF-7 cells treated with 0.25, 0.5 and 1 mg/mL of β-Nb_2_ZnO_6_ (**A**), respectively. (**e**) Untreated HCT116 cell, (**f**–**h**) HCT116 cells treated with 0.25, 0.5 and 1 mg/mL of β-Nb_2_ZnO_6_ (**A**), respectively. (**i**) Untreated HeLa cells, (**j**–**l**) HeLa cells treated with 0.25, 0.5 and 1 mg/mL of β-Nb_2_ZnO_6_ (**A**), respectively. The blue tint signifies DAPI-stained cell nuclei (200× magnification, scale bar is 50 µm for all images).

**Figure 11:**
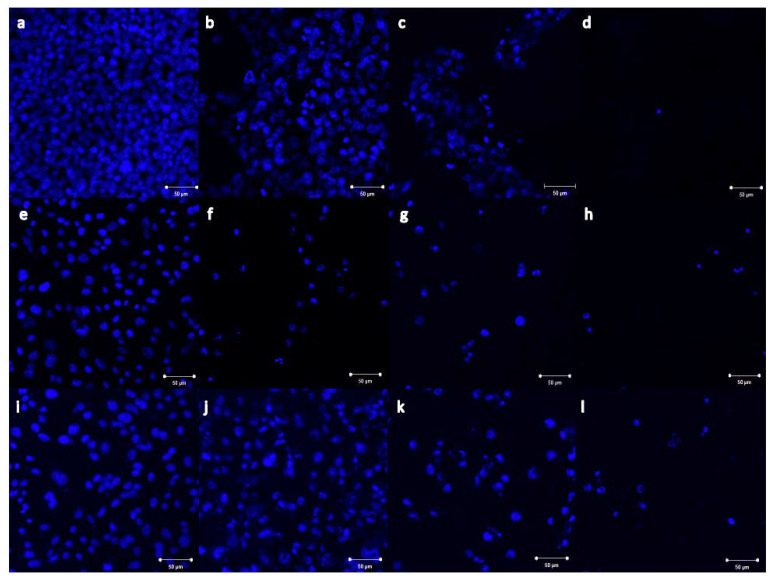
Confocal microscopic images of MCF-7, HCT116 and HeLa cells cells treated with β-Nb_2_ZnO_6_ nanoparticles (**B**). The cells were treated for 48 h with 0.25, 0.5 and 1 mg/mL of β-Nb_2_ZnO_6_ (**B**) nanoparticles. (**a**) Untreated MCF-7 cells, (**b**–**d**) MCF-7 cells treated with 0.25, 0.5 and 1 mg/mL of β-Nb_2_ZnO_6_ (**B**), respectively. (**e**) Untreated HCT116 cell, (**f**–**h**) HCT116 cells treated with 0.25, 0.5 and 1 mg/mL of β-Nb_2_ZnO_6_ (**B**), respectively. (**i**) Untreated HeLa cells, (**j**–**l**) HeLa cells treated with 0.25, 0.5 and 1 mg/mL of β-Nb_2_ZnO_6_ (**B**), respectively. The blue tint signifies DAPI-stained cell nuclei (200x magnification, scale bar is 50 µm for all images).

The morphological impacts of β-Nb_2_ZnO_6_ nanoparticles (**A** and **B**) on treated cells were indicated by imaging using confocal microscopy. MCF-7, HCT116, and HeLa cells together with 0.25, 0.5, and 1 mg/mL of β-Nb_2_ZnO_6_ (**A**) and (**B**) nanoparticles were treated for 48 h and fixed with cold absolute methanol prior to the staining procedure. The cells were stained with DAPI, a blue fluorescent dye that binds to adenine/thymine-rich segments on DNA (blue) to visualize the nuclei. Treated MCF-7, HCT116, and HeLa cells showed nuclear fragmented structures at the lowest used concentration as well as a reduction in cell number in comparison to the untreated cells. It was obvious that β-Nb_2_ZnO_6_ (**A**) and (**B**) were dramatically changing the morphology of treated cells upon increasing the concentration, as shown in Figure 10. Interestingly, β-Nb_2_ZnO_6_ (**A**) and (**B**) treated HeLa cells showed more DAPI stained cells which is consistent with the cell viability results and estimation EC_50_ that HeLa cells were weakly affected by the used concentrations after 48 h of treatment (Figure 10 and Figure 11). The imaging results clearly demonstrated a correlation with the cytotoxicity results of β-Nb_2_ZnO_6_ nanoparticles, implicating their ability in inducing morphological changes and nuclear fragmentation which might be associated with a cell death mechanism.

## 3. Materials and Methods

### 3.1. Synthesis of β-Nb_2_ZnO_6_

Niobium chloride (0.270 g) and zinc nitrate (0.2974 g) were weighed and shifted to a Teflon-lined autoclave with 20 mL of distilled water. After stirring, urea (0.2402 g) and ammonium fluoride (0.148 g) were added to the mixture. After stirring, the autoclave was heated at 200 °C for 12 h. The obtained precipitation was centrifuged and washed repeatedly using deionized water, followed by washing with ethanol, and dried overnight (60 °C). The samples were calcined at 500 °C and 700 °C in the furnace and assigned as (**A**) and (**B**), respectively.

The morphology and size of β-Nb_2_ZnO_6_ nanoparticles (**A**) and (**B**) were determined by a transmission electron microscope (TEM) (FEI, Morgagni 268, Brno, Czech Republic) and X-ray diffraction (Rigaku, Japan) quantified with Cu-Kα radiation (λ = 1.5418 Ǻ) with a 1° per minute speed of scanning (range 10–80°). Surface area (BET) was determined by Micromeritics ASAP 2020 PLUS (Norcross, GA, USA) by degassing the samples (180 °C) and by employing N_2_ adsorption data with a range of relative pressure (P/P_0_) from 0.0 to 1.0. A diffuse reflectance UV-visible spectrophotometer was used for recording the UV-Visible spectra (UV-Vis, JASCO V-750, Great Dunmow, Essex, UK).

### 3.2. Photocatalytic Activity

The aim of evaluating the photocatalytic action of the β-Nb_2_ZnO_6_ nanoparticles (**A**) and (**B**) was realized by achieving photodegradation of methyl orange under visible light irradiation using a Xenon lamp (300 W, with > 400 nm cut-off filter). Each experimental set consisted of 0.050 g of β-Nb_2_ZnO_6_ nanoparticles (**A**) and (**B**) dispersed in 50 mL methyl orange (aqueous 10 mg/L). For the establishment of an adsorption-desorption balance between the photocatalyst and methyl orange, the solution was continuously stirred for a specific period in the dark followed by illumination with the Xenon lamp. After 15 min, 3 mL of the sample was removed and centrifuged. The degradation of methyl orange was calculated at 465 nm using UV-Visible spectrophotometry. The extent of degradation was calculated by

Efficiency (%) = (C_0_ − C)/C_0_ × 100

where C_0_ is the initial methyl orange concentration and C is the time-dependent concentration of methyl orange following irradiation with β-Nb_2_ZnO_6_ nanoparticles (**A** and **B**).

### 3.3. Cell Culture and Cytotoxicity of β-Nb_2_ZnO_6_ Nanoparticles

The human cell lines (American Type Culture Collection, Manassas, VA, USA) applied to test the cytotoxicity of β-Nb_2_ZnO_6_ nanoparticles (**A** and **B**) against were: MCF-7 (breast cancer), HCT116 (human colon cancer), and HeLa (cervical cancer), the cell lines attained were as (MCF-7-ATCC^®^HTB-22™, HCT116-ATCC^®^CCL-247™, and HeLa-ATCC^®^CCL-2™, preserved with Dulbecco’s Modified Eagle’s Medium (DMEM) accompanied with 1% L-glutamine, 10% fetal bovine serum, and 1% penicillin-streptomycin (Gibco) at 37 °C with a humidity of 5 percent CO_2_. Cells (trypsinized with Trypsin-EDTA 0.25%, TFS) were rested (5 min) at 5 percent CO_2_ humidity before defusing with 1:1 of DMEM and finally placed under 1000× rpm in a centrifuge (5 min). Following three to six treatments, cells were situated in 96-well plates at 10^4^ cells/well and maintained in DMEM for 24 h. The cells were independently reacted with β-Nb_2_ZnO_6_ (**A**) and (**B**) nanoparticles (at concentrations 1, 0.5, 0.25, and 0.125 mg/mL) with a dilution of 1 mL DMEM and 100 mL of each of the above was used in replicates of two post removal of precultured media and incubation period of 24, 48, and 72 h.

The viability check was done two times by addition of MTT solution (Sigma) prepared as 5 mg/mL in 1 × phosphate-buffered saline. MTT (10 µL solution) was placed into each well as well as positive controls with 0.5 mg/mL concentration. The assay was carried out for 4 h at 37 °C at the end of which, 100 µL of dimethyl sulfoxide (DMSO) was introduced to bring about the conversion of tetrazolium salts to formazan by metabolically active cells. Spectra at 570 nm were noted with the help of SYNERGY Neo2 multi-mode microplate reader, Biotek, to compute cell viability as

Cell viability (%) = Abs_sample_/Abs_control_ × 100

MTT cell viability results were stated as the mean ± standard deviation (SD) of two objective experiments. The results were subjected to the ordinary two-way ANOVA test which was carried out by GraphPad Prism Software (GrapPad, La Jolla, CA, USA). In all cases, *p*-value ≤ 0.05 was considered significant (GP: 0.1234 (ns), 0.332 (*), 0.0021 (**), 0.0002 (***), <0.0001 (****).

### 3.4. Imaging by Confocal Microscopy

MCF-7, HCT116, and HeLa cell lines were seeded in 8-well Nunc™ Lab-Tek™ Chamber Slide System (Thermo Fisher Scientific) at 35 × 10^4^ cells/well together with 0.25, 0.5, and 1 mg/mL of each β-Nb_2_ZnO_6_ (**A**) and (**B**) nanoparticles for 48 h. The cells were fixed using cold absolute methanol for 10 min, then treated with ProLong™ Gold Antifade Mountant plus DAPI (4′,6-diamidino-2-phenylindole) from TFS. The illustrations were recorded with an LSM 700 confocal microscope (Zeiss, Jena, Germany).

## 4. Conclusions

β-Nb_2_ZnO_6_ nanoparticles were effectively designed with nanoplate-like morphology using a hydrothermal method. The sample calcined at 500 °C (**A**) exhibited an improved photocatalytic action for the degradation of methyl orange compared to the sample calcined at 700 °C (**B**). The comprehensive results conclude that sample A is considered more effective on account of enhanced charge carrier separation along with synergistic repressed recombination of electron-hole pairs, making it an ideal photocatalyst. Finally, we examined the cytotoxicity of β-Nb_2_ZnO_6_ (**A**) and (**B**) nanoparticles at gradual concentrations (1, 0.5, 0.25, and 0.125 mg/mL) against MCF-7, HCT116, and HeLa cells. The results revealed that β-Nb_2_ZnO_6_ (**A**) and (**B**) nanoparticles exhibited high cytotoxic activity against MCF-7 and HCT116 cells and lower cytotoxic activity towards HeLa cells post 24 and 48 h, which turned to high cytotoxic activity after 72 h. Overall, the EC_50_ values were similar for both (**A**) and (**B**) against each tested cell line with obvious variations between MCF-7 or HCT116 and HeLa cells.

## Data Availability

Not applicable.
